# The culture of research communication in neonatal intensive care units: key stakeholder perspectives

**DOI:** 10.1038/s41372-021-01220-5

**Published:** 2021-10-18

**Authors:** Jennifer Degl, Ronald Ariagno, Judy Aschner, Sandra Beauman, Wakako Eklund, Elissa Faro, Hiroko Iwami, Yamile Jackson, Carole Kenner, Ivone Kim, Agnes Klein, Mary Short, Keira Sorrells, Mark A. Turner, Robert Ward, Scott Winiecki, Christina Bucci-Rechtweg, International Neonatal Consortium

**Affiliations:** 1 Speaking for Moms and Babies, Inc., Mahopac, NY USA; 2 Stanford University, Palo Alto, CA USA; 3 Hackensack Meridian Health, New York, NY USA; 4 CNS Consulting/National Association of Neonatal Nurses, Albuquerque, NM USA; 5 Pediatrix Medical Group of TN/National Association of Neonatal Nurses, Nashville, TN USA; 6 Carver College of Medicine, University of Iowa, Iowa City, IA USA; 7 Osaka City General Hospital, Osaka, Japan; 8 Nurtured by Design, Sugar Land, TX USA; 9 Council of International Neonatal Nurses, Inc., The College of New Jersey, Ewing, NJ USA; 10 U.S. Food & Drug Administration, Silver Spring, MD USA; 11 Health Canada, Ottawa, ON Canada; 12 Eli Lilly & Co, Indianapolis, IN USA; 13 NICU Parent Network, Madison, MS USA; 14 University of Liverpool, Liverpool, UK; 15 University of Utah, Salt Lake City, UT USA; 16 Novartis Pharmaceuticals Corporation, East Hanover, NJ USA

**Keywords:** Paediatrics, Medical research

## Abstract

**Objective:**

To assess the perspectives of neonatologists, neonatal nurses, and parents on research-related education and communication practices in the neonatal intensive care unit (NICU).

**Study design:**

Questionnaire circulated through interest groups and administered using the internet.

**Results:**

323 respondents responded to the survey. 52 were neonatologists, 188 were neonatal nurses, and 83 were parents of NICU graduates. Analysis was descriptive. Differences were noted between stakeholder groups with respect to whether current medications meet the needs of sick neonates, research as central to the mission of the NICU, availability of appropriate education/training for all members of the research team, and adequacy of information provided to parents before, during, and after a research study is completed.

**Conclusion:**

Engagement of nurses and parents at all stages of NICU research is currently suboptimal; relevant good practices, including education, should be shared among neonatal units.

## Introduction and statement of the problem

Technological and scientific advances have significantly improved neonatal outcomes over the past several decades. While the field has progressed, prematurity remains the leading cause of infant mortality worldwide and results in thousands of annual admissions to neonatal intensive care units (NICUs) along with substantial health care expenditures [1]. Despite legislative efforts to promote drug development in the pediatric population, policies incentivizing research have failed to entice manufacturers to invest in neonatal therapeutics. Further, policies obligating manufacturers to conduct research are tied to adult investigational drugs and biologic products whose mechanisms of action may play no role in addressing neonatal conditions and therefore will not serve the needs of neonates [2]. As a result, preterm neonates are routinely exposed to multiple drugs that have not been researched adequately and have not been approved by regulatory agencies for their intended use [3, 4]. This translates to the use of drugs that have not been sufficiently tested for safety, dosing, or effectiveness in this population [5]. The last class of drugs approved for use in preterm neonates that significantly impacted survival and outcome was pulmonary surfactant for respiratory distress syndrome [6].

Since it is widely recognized that the study of new and existing drugs for use in neonates has lagged behind other populations, there is a critical need to facilitate the conduct of neonatal clinical trials. While there is a critical need to innovate, participation in neonatal clinical trials has been viewed by many as ethically challenging, too risky, burdensome for parents, and as a favor provided by altruistic families to future generations [7, 8]. Practices regarding the design and conduct of neonatal clinical trials, including multi-stakeholder involvement, have been identified that may help to overcome some of these challenges [7].

The Critical Path Institute’s (C-Path) International Neonatal Consortium (INC) has united stakeholders from around the globe with a shared aim of accelerating the development of safe and effective therapies for neonates [9]. INC includes parents, neonatal advocacy organizations, neonatologists, clinical pharmacologists, neonatal nurses, regulators, and representatives from the pharmaceutical industry. INC’s primary focus is on developing practical tools and processes to facilitate the conduct of ethical and efficient neonatal clinical trials. The literature describes communication in general on neonatal units [10–15] and challenges posed to parents and staff by research [16–21]. This literature includes some suggestions about how to improve communication in general but does not report on the application to neonates of insights from other specialties [22–25]. Accordingly, INC identified a need for guidance about how to communicate with nurses and parents about clinical trials and noted that education about research underpins effective communication. Nurses and parents can play pivotal roles in ensuring the quality and efficiency of research design, implementation, recruitment, ascertainment, provision of informed parental consent, and disclosure of research results [7]. INC noted that guidance about these roles for nurses, and for reference by other professions would be useful. However, guidance about these roles cannot be compiled because, to date, there is a paucity of literature addressing research-related education and communication practices in NICUs as a critical component of recruitment and retention strategies for neonatal clinical trials. Additionally, at the initiation of this survey, there had been no other surveys that had evaluated, in parallel, the perspectives of neonatologists, NICU nurses, and parents on these topics.

We surveyed three key stakeholder groups—neonatologists/physician researchers (“neonatologists”), neonatal nurses/nurse researchers (“neonatal nurses”), and parents of NICU graduates (“parents”)—to assess personal perspectives on research-related education and communication practices in NICUs located across the globe. We evaluated stakeholder perspectives on the role of research in advancing neonatal care in the NICU, the current clinical and research communication flow in the NICU, education and training of neonatal personnel about the value of neonatal research, the research consent process, and disclosure of study results to families. Having knowledge of these perspectives may inform and enhance existing NICU communication strategies, improve engagement of neonatal staff and parents in discussions regarding neonatal clinical trials, and increase parental understanding of and participation in neonatal research [26].

## Methodology

The questionnaire was developed using a stepwise consensus approach [27] with input from multiple relevant stakeholders (neonatologists, nurses, regulators, parents, and pharmaceutical industry representatives) to assess research-related communication practices in NICUs globally. Independently, three of the stakeholder groups (neonatologists, nurses, and parents) conducted a targeted review of literature relevant to their respective roles to assess the availability of and insights related to research communication practices in the NICU. Keywords included: family-centered care, consent, culture of research, clinical trials, research disclosure, communication, NICU, newborn, neonate, family, education, organizational structure, interprofessional relations, results reporting, and clinical trials coordinator. Approximately 30 articles were identified and evaluated as part of the independent review.

While the literature search found studies on the informed consent process and principles of family-centered care, it exposed a paucity of available information on communication practices regarding research disclosure, flow of communication in NICUs, strategies to communicate essential information across care teams, and training of neonatal personnel specific to the conduct of clinical trials. These findings led the research team to identify six domains for further evaluation. These included: (1) role of research in the NICU; (2) education and training of NICU personnel about the role of research; (3) engagement of parents of NICU graduates in study design and education; (4) current NICU communication flow; (5) research consent processes; and (6) research results disclosure. Representatives from the relevant stakeholder groups contributed to survey content development for each domain. Survey development then merged questions from two of the original domains (e.g., engagement of parents of NICU graduates in study design, and education/training of NICU personnel) into one final domain called education and training of neonatal personnel on the role of neonatal research, heretofore called “education and training.” This resulted in five final survey domains.

The survey used branching to direct the respondents to specific paths based on the particular stakeholder cohort to which they belonged. Each respondent provided individual anonymized responses. With the exception of specific demographic questions, survey questions for neonatologists and neonatal nurses were identical. Due to an error in the skip logic in the survey, some questions were addressed only to neonatal nurses/nurse researchers. Questions assessing parent perspectives were modified using lay language to enhance readability. The survey employed questions utilizing Likert-type, multiple choice, and binary responses. Skip logic was employed throughout the survey depending on how respondents rated their agreement with a statement. The length of the survey varied with more questions posed to medical professionals compared to parents. The survey was piloted for response content and platform usability.

The survey launched in August 2018 and closed in November 2018 using the Survey Monkey^®^ [28] platform (cloud-based software) from a link provided on the C-Path INC web landing page. Initially, invitations with the survey link were sent via email to a convenience sample of INC members who then disseminated the invitation to relevant organizations and interested individuals via emails, listservs, or social media. Participation was voluntary and without compensation. The survey study was determined to be exempt from review by an institutional review board.

Survey results were compiled using analytics software within Survey Monkey^®^ [28] and reported using descriptive statistics (including frequency distribution, mean, median). This was an exploratory, scoping study with a large number of potential comparisons. Accordingly, statistical testing of comparisons between groups was not planned nor conducted. Investigators reviewed and analyzed survey results in a series of meetings designed to identify key findings within each domain, which are described below.

## Results

### Demographics

Among the 323 respondents who entered the survey, 52 were neonatologists, 188 were neonatal nurses, and 83 were parents of NICU graduates. While the majority of nurses and parents were from the United States, the neonatologists were evenly represented across the United States and Europe. Most responding nurses and parents held a bachelor’s degree or higher. The majority of medical professionals defined their NICU’s level of care as 3 or 4. While the majority (69%) of neonatologists indicated that they engage in both patient care and research at their institution, only 17% of nurses indicated that their professional role includes both patient care and formal research responsibilities. There was wide variability among parents between the time their child was cared for in the NICU and their participation in the survey. Table 1 provides details on the demographics of the survey participants.

**Table 1:** Demographics of the survey participants.

	Neonatologists (*n* = Respondents/responses) %	Neonatal Nurses (*n* = Respondents/responses) %	Parents of NICU graduates (*n* = Respondents/responses) %
Number of Survey Respondents	(*n* = 52/52)	(*n* = 188/188)	(*n* = 83/83)
Region	(*n* = 52/52)	(*n* = 188/188)	(*n* = 83/83)
United States	38.5	78.7	66.3
Europe and Switzerland	40.4	9.6	21.7
Japan	9.6	3.2	1.2
Canada	1.9	3.2	2.4
Other	9.6	5.3	3.6
Education	(*n* = 48/48)	(*n* = 176/176)	(*n* = 75/75)
Bachelor’s degree or higher	100	95.5	69.3
Master’s or higher	100	62.5	37.3
NICU Level of Care^a^	(*n* = 42/45)	(*n* = 172/191)	NA
Level 1 or 2	6.7	17.8	NA
Level 3 or 4	88.9	80.6	
Unsure	4.4	1.6	
Research Role in NICU^b^	(*n* = 39/43)	(*n* = 167/176)	NA
Member of designated research team	16.3	8	NA
Direct patient care only	2.3	23.3	
Patient Care and Research	62.8	16.5	
Direct patient care (Informal research responsibilities)	2.3	40.3	
Other	16.3	11.9	
Age when child was cared for in NICU	NA	NA	(*n* = 74/74)
Between 25 and 34 years	NA	NA	63.5
Between 35 and 44 years			27
Time between child in NICU and Survey	NA	NA	(*n* = 72/72)
Within last 12 months	NA	NA	12.5
Between 12 months and 2 years			18.1
Between 2 and 5 years			30.5
Greater than 5 years			38.9

^a^Level 1 (Definition: newborn care for babies at low risk, e.g., newborn nursery). Level 2 (Definition: specialty care for stable or moderately ill newborns born > 32 weeks gestation who are born with problems that are expected to resolve rapidly). Level 3 (Definition: specialty care for newborns who are born at <32 weeks gestation, weigh <1500 g at birth, or have medical or surgical conditions necessitating complex care). Level 4 (Definition: include the capabilities of a Level 3 nursery with additional capabilities in the care of the most complex and critically ill newborns, and have pediatric medical and pediatric surgical specialty consultants continuously available 24 h a day).

^b^Where respondent and response values differ, percentages reported are the percent of the responses.

### Research in the NICU

The survey assessed the role of research in the NICU (see Table 2). The majority of respondents in all groups reported that standard approaches to care are used and can improve care. All groups strongly agreed that research protocols are needed to develop standard approaches to care. All respondent groups were very likely to say that research should be central to the work of a NICU.

**Table 2:** Stakeholder perspectives on available medications and the role of research.

	Disagreed/Strongly Disagreed			Agree/Strongly Agree			Neutral/Unsure		
Neonatologists *n* = 37, Nurses *n* = 135, Parents *n* = 54 unless otherwise indicated by reference to the table footnote.	Neo-natologists (%)	Nurses (%)	Parents (%)	Neo-natologists (%)	Nurses (%)	Parents (%)	Neo-natologists (%)	Nurses (%)	Parents (%)
Stakeholder Perspectives on Available Medications and the Role of Research									
Current medications are sufficient	82	36	31	5	41	47	13	23	23
Studies conducted by pharmaceutical companies are needed	11	14	20	72	55	58	18	31	22
“Academic” studies (not conducted by pharmaceutical companies) are sufficient	66	34	27	18	33	47	16	33	27
My NICU uses a standard approach to neonatal care	5	6	8	79	84	67	16	10	25
Standard approaches to neonatal care improve neonatal outcomes	3	2	10	89	94	63	8	4	27
Research protocols are required to improve a baby’s outcome	5	2	0	95	95	90	0	3	10
Research is a central component of my NICU’s work	3	24	17	76	55	50	21	21	33
Research should be a central component of a NICU’s work	8	4	3	82	82	83	8	14	14
There are sufficient protections for newborn enrolled in a research study	8.1	2.2	13	81	71	44.4	10.8	24.6	42.6
Stakeholder Perspectives about Special Protections in Neonatal Research									
Routine adverse event reporting is used to identify safety concerns in neonatal studies	2.7	1.4	3.7	91.9	88.8	70.3	5.4	9.6	26
Bedside clinicians who are NOT on the research team contribute to the oversight of NICU studies	2.7	4.44	7.4	86.4	88.2	66.7	10.8	7.3	25.9
No special protections are provided^a^	78.3	77.7	55.1	10.8	3.8	8.1	10.8	18.4	36.7
Scientific and ethical review of protocols by neonatal experts provide assurance that benefits of study participation outweigh risks^a^	5.4	4.6	10.2	75.6	78.4	57.1	18.9	16.9	32.6
There are special protections to minimize risk, burden (e.g., limiting the amount of blood drawn) and discomfort of neonates in studies^a^	0	3.1	4.1	86.5	76.9	67.3	13.5	20	28.5

^a^MD *N* = 37, RN *N* = 130, parent *N* = 49.

A series of questions was asked about respondents’ awareness of special protections that exist for studies involving sick newborns (Table 2). The majority of respondents from all three groups recognized that special ethical protections, ethics review boards, data monitoring committees, and risk minimization procedures (e.g., limited, low volume blood draws) provide protection for neonates involved in research. When parents were asked if sufficient protections exist to ensure the rights and safety of their babies when enrolled in a research study, 44% agreed while 42.6% were unsure.

**Fig. 1:**
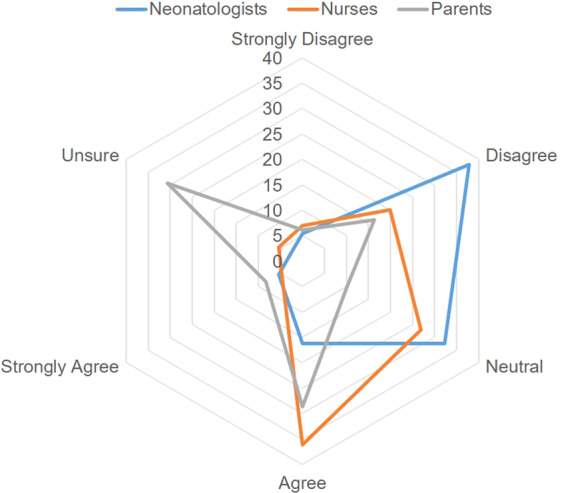
Radar plot of stakeholder perspectives about preclinical research. The proportion of respondents from each stakeholder group is shown for the prompt that “preclinical research provides an assessment of safety that is adequate to proceed to conducting a drug study in neonates”. The responses by Neonatologists (n = 37) are shown in a blue line. The responses by Nurses (n = 130) are shown in an orange line. The responses by parents (n = 49) are shown in a grey line.

One-third of all three stakeholder groups expressed an unsure/neutral response that “pre-clinical research using animal models” provides an assessment of safety that is adequate to proceed to conducting a drug study in neonates. (Fig. 1).

### Current NICU communication flow

Parental presence, interaction with the medical team, and assistance with the care of the neonate were acknowledged by both neonatologists and nurses to be common practice in their NICUs. Neonatologists and nurses indicated that their institutions offer prenatal visits to the NICU and interactions with staff for high-risk pregnancies. Similarly, the majority of neonatologists (78%) and nurses (87%) noted that their institutions have a standard approach to prenatal consultations involving at least one member of the neonatal healthcare team. Survey respondents agreed that sufficient flexibility exists to allow parents and various members of the neonatal and obstetrical health care team to request a prenatal consultation. However, 20% of parents indicated that they were “Never” or “Rarely” offered meetings with the neonatal team during the prenatal or postnatal period.

Perspectives in the communication that surrounds decision-making in NICUs (both clinical care and research) were also explored. The majority of the nurses (78%) and physicians (74%) felt that families are included in decision-making processes in their NICU, with 91% of nurses and 92% of physicians indicating they felt free to advocate for their patients during the decision-making process. When specifically asked if this included speaking out to other staff, 86% of nurses and 67% of physicians reported being able to question the decision of those with more authority or experience.

Thirty-seven percent of neonatologists reported that their NICU has a system in place to inform families about upcoming research compared to ongoing (53%) research. Just 21% of nurses reported that their NICU had a system in place to provide updates about an ongoing neonatal research study. In NICUs identified to have systematic approaches to communication about upcoming or ongoing research, neonatologists and nurses identified “specific meetings to discuss research,” “contact by research staff,” and “staff meetings” as “Effective” or “Very Effective” communication strategies. Neonatologists and nurses both identified that these approaches were more effective in informing staff about ongoing research compared to posted study notices or bedside rounds.

### Education and training of NICU personnel on the role of research

All surveys asked whether the respondent’s institutional education and/or training programs include families whose sick neonates participated in the research. The majority of parents (70%) noted that the institution where their baby was treated “Never” involved families in research education or training programs for current NICU parents. When the professionals were asked this question, 29% of neonatologists and 39% of nurses responded that their institutions did not include families in their training programs. In addition, most neonatologists (83%) and nurses (58%) indicated that training on topics relevant to research in the NICU is available at their institution, such as the need for research and research consent processes. However, of those nurse respondents who indicated that training is available, the perception was that this training was not available to all members of the team responsible for the patient enrolled in the research study (42%).

Due to an error in the skip logic programming, only nurses who indicated that their institution provided training on research topics were asked a series of additional questions as to by whom or how the training was provided. The most frequently offered methods of research training for nurses included informal coaching (46%), online materials (40%), and reading materials (40%). Training for nurses was frequently led by research nurses or study coordinators (52%) followed by research physicians (37%) and neonatologists (36%). Nurse respondents who indicated that their institution provided research training agreed that “special measures to protect the rights of the neonatal study participants” (84%) and “psychological and psychosocial aspects related to obtaining parental consent” (70%) are covered. Fewer respondents agreed that the “history/role of drug development in neonates” (57%) and “phases of drug development” (55%) are included in the training. In addition, about one-third were either “Neutral” or “Unsure” that these concepts were included.

Nurses were also asked about additional topics related to neonatal research that was included in their professional education, especially in “academic settings”. More than half indicated that they had received education regarding the “role of research in improving care and establishing new and effective treatments” (58%), how to “critique research results” (54%), and how to “evaluate clinical study design” (50%). Only 10% of nurses reported that they received education on “drug development processes” or “design and conduct of drug development research.” Approximately 25% of nurses reported that their formal, pre-qualification training did not include any of these topics.

Nurses’ perceptions on the effectiveness of their education about research were also evaluated. More than half the nurses (53%) indicated “Neutral,” “Unsure,” or “Somewhat Effective” regarding the effectiveness of their professional education or hospital training in informing their ability to participate in neonatal research.

### Research consent process

A small proportion of neonatologists and nurses noted that consent for study participation is “Usually” (23%) or “Always” (14%) sought from families before the birth of a potentially sick neonate or in advance of an anticipated event in the NICU (28% vs 21%, respectively). A large segment of respondents was “Unsure” about the consent process in their NICU, including whether discussions on research in the NICU were included in all antenatal consultations, whether certain consent practices (continuous consent, affirmation of research consent) were offered in their NICUs, and about the timing for consent in relation to antenatal consultations.

Among parents, 45% had been asked to consider enrolling their neonate in a research study with 18 of the parent respondents (37%) noting that they provided informed consent. Of these 18 parents, the majority “Agreed” or “Strongly Agreed” that they felt comfortable asking questions before they agreed to consent, received information that was adequate to make a decision about consent, and had enough time to consider whether their baby should participate in the study. However, 33% of the parents who consented noted it was not clear where they could obtain more information about the study once their baby was actively participating.

### Disclosure of research results

**Fig. 2:**
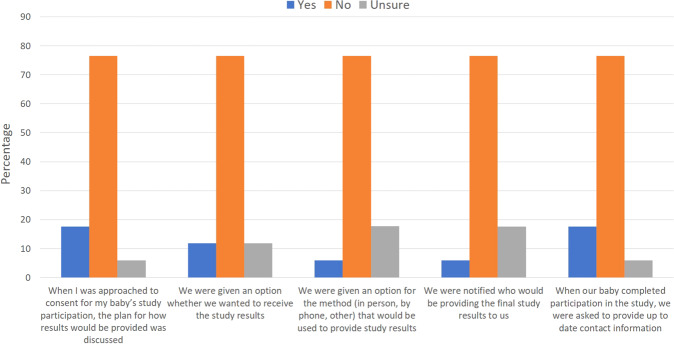
Disclosure of research results. Parent reports of how disclosure of results was handled during and after recruitment to a study. This figure includes responses from 17 parents.

Although the majority of respondents “Agreed” or “Strongly Agreed” that study results should be made available to families whose neonates have participated in research when the study is completed, 25% of neonatologists provided a “Neutral” response. Additionally, the majority of respondents understood that research results were required to be made public. However, both neonatologists and nurses noted that the process of informing parents whose neonates were enrolled in clinical research about study results is inconsistent. Fewer than 30% of neonatologists and nurses stated that their institution has a standard process to communicate results after study completion. Additionally, 60% of neonatologists and 35% of nurses agreed that their institution has a standard approach in place to maintain up-to-date contact information for parents after study completion. Among parents who had consented for their baby to participate in a clinical trial, most noted that they were not informed/advised about the plan for results disclosure, nor was their preference sought on the method of disclosure (Fig. 2).

## Discussion

Our survey of 323 respondents from three distinct and synergistic stakeholder groups in neonatal clinical research begins to address some of the gaps in our understanding of effective research-related communications practices relevant to the development of guidelines intended to contribute to the successful recruitment and conduct of future clinical trials in NICUs. To our knowledge, this is the first description and comparison of the perceptions of NICU parents, nurses, and physicians to a set of parallel survey questions regarding communication, across multiple domains, that are relevant to the role of research in the NICU. Improving stakeholder engagement and understanding of the importance and unique challenges of research in neonates requiring intensive care are important goals given the lack of evidence behind many therapies commonly used in the NICU. Establishing best practices for research-related communication will build on existing good practice for communication in general by extending the content of communication but also the methods of communication [10, 11, 13–15, 29].

In general, physicians felt that existing drugs used routinely in the NICU are not adequate to meet the needs of neonates. The majority of nurses and parents felt that currently available drugs were adequate. We note that 33% of nurses did not agree that studies of new and existing drugs sponsored by pharmaceutical companies are important. The perceptions of some parents and nurses may be a barrier to research about drugs. Respondents valued a standard approach to care. The proportion of respondents who agreed/strongly agreed that research protocols are needed to develop standard approaches to care (greater than 90% of respondents in all three groups) was greater than the proportion that reported standard approaches to care were utilized in their NICU. This suggests that there is an appetite for research among all groups. More than 80% of respondents in all groups reported that research should be central to the work of a NICU. Respondents in all groups were less likely to report that research actually was central to their NICU. While 75% of professionals were aware that there are extensive protections for neonates who participate in clinical trials, fewer agreed that consideration of data from pre-clinical or animal studies is adequate to proceed to a clinical study in neonates. The data suggest that understanding the role of preclinical research for neonatal conditions is an educational need in the neonatal community. Thus, communication needs to include staff awareness of the broader issues and the ability to share information about those issues with parents. This is in contrast to other work on communication that focuses on the “here and now” of the parents’ experiences [10, 11, 13–15, 29] or has focused on training physicians about research [30].

Routine communication practices in the NICU provide a benchmark for better understanding the need for improvements in research communication strategies. While a large proportion of respondents reported that families were briefed about neonatal care antenatally, only half reported that their institutions had systems in place to inform families about existing research. This was reflected in the small proportion of parents who reported having heard about research studies before delivery. In addition, 20% of families reported that they had not been offered routine communication about neonatal care so that the scope for increasing opportunities to communicate with parents about studies (especially during antenatal consultations) may be limited by the extent of routine communication practices in some NICUs. Most importantly, less than 20% of nurses reported that they were able to provide their unique input into the preparation of research protocols that would be conducted in their NICUs (e.g., a nurse champion being appointed for each individual research study to advocate for other nurses and families), despite this being recognized as good practice [5]. While neonatologists were aligned with nurses and parents on many topics (such as the importance of consent in clinical research), neonatologists were more likely to report that research-related communication was available on their unit than nurses or parents. Neonatologists and NICU management should review whether communications that are visible to neonatologists are visible to, or useful for, nurses and families.

Optimal planning and conduct of neonatal clinical trials is far from universal. Respondents from all stakeholder groups reported limited efforts to inform and engage parents in research efforts during their pregnancy. While fewer than half of parents had been approached about research, the majority (but not all) of those approached reported that they had enough time to consider the issues. There are clearly considerable opportunities to inform more parents about research: this will need careful attention to the practicalities of seeking consent in emergency situations, such as newborn resuscitation. The need to share results of studies was acknowledged by all stakeholder groups. The limitations seem to be in the proactive planning of research results dissemination prior to the initiation of a study.

Inadequate education and training about research could explain the findings relating to understanding, communication, and involvement of nurses in research-related processes. A substantial proportion of professionals were not aware of training about obtaining informed consent in their institutions. Some, but not all, training included important aspects of drug development processes. Key findings were: (1) nearly half of nurses reported that training was not available to all relevant members of the team that conducts research and; (2) more than half of nurses felt that the available training was not effective in supporting their contribution to research studies. There is clearly considerable scope for improving the quantity and quality of education and training about research among neonatal nurses, who are the crucial interface between families and the research enterprise. Other specialties have described the value of taking a comprehensive approach to improving the quality of work in critical care [31]. Our findings suggest that there is a need to improve education about research across all neonatal health care professions.

Key limitations of this survey were the nature of the scoping exercise which was intended to identify key themes for future work, the lack of cognitive testing of the survey instrument, the relatively small dataset that does not allow for more granular analysis of the results, and the survey structure which incorporated skip logic and was lengthy. The length may have contributed to the noticeable attrition in responses later in the survey. Additionally, the survey was accessible via a public landing page to allow for a diverse set of respondents (i.e., clinicians and researchers) to participate. This modality did allow for two trainees to self-identify and contribute data as part of the neonatologist cohort. Further, an error in skip logic in the education and training section of the medical professionals survey only allowed for nurses to respond within that set of domain questions. Descriptive analysis informed thematic analysis that guided the messages presented in this paper.

The study was designed using the principles of qualitative research so that statistical testing to compare groups was not performed. However, some differences observed in the responses between groups may warrant further study. Specifically, these results lead us to hypothesize that nurses are not included in research adequately despite their commitment to, and involvement in, research during everyday clinical practice. Also, we speculate that increased education about the nature and benefits of clinical trial design and their implementation will be useful, particularly for nurses.

This survey was disseminated via INC to active neonatal networks within each stakeholder group and did not have a population sampling frame. Within the parent respondents, we saw a highly educated parent sample which may not be representative of the general NICU graduate parents. Most respondents had experience in Level 3 and 4 NICUs, with neonatologists having significant research experience, whereas only 25% of nurses had formal research responsibilities in their units. This is unfortunate, given that neonatal research depends on extensive work at the bedside and with families, done as part of routine duties by nurses in the NICU. This is recognized by the 42% of nurses who reported informal involvement in research. The lack of formal engagement of nurses in ensuring trial success is part of the untapped potential for most NICUs whose objectives include research. While the survey results are not necessarily generalizable, data nevertheless provide key themes to inform future work to improve research in NICUs. Even though the findings are likely to reflect a “positive” bias in favor of research among this self-selecting sample, results still indicate key knowledge gaps and numerous opportunities to standardize approaches and develop a comprehensive education program for all stakeholder groups. This should ultimately result in improved research processes and greater parental participation in clinical research.

The implications of these results are that:

1. The barriers and delays in the conduct of neonatal clinical trials may be reduced if health care professionals and families have a greater understanding of the specific need for, and nature of, neonatal research.
2. It is essential to increase the involvement of nurses and parents in all stages of research.
3. Good practices in research design, including informed consent plans and dissemination of results, should be shared among neonatal units.

## Conclusion

The survey results suggest that there are considerable opportunities for improving communication with parents and nurses about clinical trials on neonatal units. Further work is urgently needed to define, standardize, and implement appropriate interventions relating to:

(A) Information about the need for research about drugs used in neonatal care and opportunities to join research studies.
(B) Education for nurses about research.
(C) Recognition of nurses’ contributions to research.
(D) Best practice about trial recruitment in NICUs, including parental consent.
(E) Ensuring parents receive information about the results of any completed studies to which their baby was recruited.

We hope that these findings lead to further research that leverages the involvement of all stakeholder groups in order to conduct appropriate clinical research and improve care for vulnerable neonates.
